# Using Personal Sensors to Assess the Exposome and Acute Health Effects

**DOI:** 10.3390/ijerph110807805

**Published:** 2014-08-06

**Authors:** Mark J. Nieuwenhuijsen, David Donaire-Gonzalez, Maria Foraster, David Martinez, Andres Cisneros

**Affiliations:** 1 Centre for Research in Environmental Epidemiology (CREAL), Barcelona 08003, Spain; E-Mails: ddonaire@creal.cat (D.D.-G.); mforaster@creal.cat (M.F.); dmartinez@creal.cat (D.M.); 2 CIBER Epidemiología y Salud Pública (CIBERESP), Madrid 28029, Spain; 3 Universitat Pompeu Fabra, Barcelona 08003, Spain; 4 Physical Activity and Sports Sciences Department, Blanquerna Foundation, Barcelona 08022, Spain; 5 Ateknea Solutions, Barcelona 08940, Spain; E-Mail: Andres.cisneros@ateknea.com

**Keywords:** exposome, sensors, air pollution, green space, noise, temperature, UV, physical activity, smartphone, geolocation, mobility

## Abstract

*Introduction*: The exposome encompasses the totality of human environmental exposures. Recent developments in sensor technology have made it possible to better measure personal exposure to environmental pollutants and other factors. We aimed to discuss and demonstrate the recent developments in personal sensors to measure multiple exposures and possible acute health responses, and discuss the main challenges ahead. *Methods*: We searched for a range of sensors to measure air pollution, noise, temperature, UV, physical activity, location, blood pressure, heart rate and lung function and to obtain information on green space and emotional status/mood and put it on a person. *Results and Conclusions*: We discussed the recent developments and main challenges for personal sensors to measure multiple exposures. We found and put together a personal sensor set that measures a comprehensive set of personal exposures continuously over 24 h to assess part of the current exposome and acute health responses. We obtained data for a whole range of exposures and some acute health responses, but many challenges remain to apply the methodology for extended time periods and larger populations including improving the ease of wear, e.g., through miniaturization and extending battery life, and the reduction of costs. However, the technology is moving fast and opportunities will come closer for further wide spread use to assess, at least part of the exposome.

## 1. Introduction

The exposome encompasses the totality of human environmental (*i.e.*, non-genetic) exposures from conception onwards, complementing the genome [1,2]. The concept of the exposome and how to assess it has led to lively discussions with varied views [3,4,5,6,7,8,9]. Although at this stage it may not be possible to measure or model the full exposome, some recent European projects such as HELIX [9,10], EXPOsOMICS [11], and HEALS [12] and the American initiative HERCULES [13] have started to make first attempts. 

Environmental exposures such as air pollution [14,15,16], temperature [17] and noise [18] have been associated with adverse health effects, while UV [19] and green space [20] have been associated with both positive and negative health effects, and are therefore important to measure. Blood pressure is related to mortality and a large burden of disease [21], while heart rate variability [15] and lung function [22] are important health parameters. 

Recent developments in sensor technology have made it possible to better measure, e.g., personal exposure to environmental air pollution [23]. Further developments are ongoing as part of the NIEHS exposure biology programme [24]. Also, large European projects such as ICEPURE (UV) [25], TAPAS (location, physical activity) [26], PHENOTYPE (location, physical activity, mood) [27], EXPOsOMICS (location, physical activity, air pollution) [11], HELIX (location, physical activity, UV, black carbon) [10] and CITI-SENSE (location, physical activity and air pollution) [28] are developing and applying sensors and software to assess personal exposure for health research. Recent publications showed the use of smartphones to obtain information on mobility to estimate inhaled air pollution doses [29] and physical activity [30], accelerometers to obtain physical activity [31], while others have used GPS and small sensors to measure mobility, air pollution and noise [32,33,34,35,36,37]. 

Furthermore the improvements and miniaturization of equipment to measure health parameters such as lung function [38], blood pressure [39] and heart rate variability [16] have opened up the possibility to measure environmental exposures and health simultaneously to assess the effects of short term exposures on acute responses, which may contribute to chronic health effects. 

We aim to discuss and demonstrate the recent developments in personal sensors to measure multiple environmental exposures, and discuss the main challenges ahead, by putting together a personal sensor set that measures a comprehensive set of personal environmental exposures continuously over 24 h to assess part of the current exposome and acute health responses, which in this case are mainly physiological responses as markers for health.

## 2. Methods and Results

We focused on the main environmental outdoor exposures and searched and found a range of sensors to measure air pollution, noise, temperature, physical activity, location, emotional status/mood, blood pressure, heart rate and lung function and to obtain information on green space, and mood (Table 1) and put it on one person. We searched in PubMed, ScienceDirect and used the Google search tool to search the internet. The main criteria for choosing and using the resources were that they measured the main environmental exposures of interest, the relative ease of use, that that they could be carried by a person and that they measure 24 continuously or make repeated assessments. The smartphone which measured location, physical activity, and altitude using a built in App, and was used to take photos of green space, was worn on a SPIbelt around the waist. The air pollution (including the extra battery) and noise sensors were placed inside a small back pack and the sensor to measure temperature and relative humidity on the side of the bag pack. Most sensors measured continuously, but blood pressure and lung function were measured every two hours during waking hours, and green space, only when it occurred. Emotional status/mood was assessed at random by sending a short question. The initial idea was to collect and process the data from the various sensors through a smartphone, but finally this was only possible with data collected through the App or photos, and the UV sensor via Bluetooth. The other data were downloaded and synchronised afterwards.

**Table 1:** Personal sensor set characteristics.

Instrument	Measure	Manufacturer	Cost (Euros)	Battery Life/Memory		Recording Resolution		Weight	User Comments
eMotion FB130397	HRV	Mega Electronics Ltd., Finland	590	8 days		1000 Hz		16 g	Easy to wear Difficult with showering
Omron M10-IT	Blood pressure	OMRON Healthcare, The Netherlands	70	84 readings per user		Not applicable		660 g	Easy to use Not always possible to do at set times
Piko-1	Lungfunction FEV1-PEF	nSpire Health, USA	70	96 readings		Not applicable		35 g	Easy to use Not always possible to do at set times
Smartphone Galaxy S3 (GT-I9300)	Photos green space	Samsung, Korea	350	24 h *		Not applicable		213 g	Easy to wear in SPIbelt, easy to forget to take photos
EMA, SMS	Emotional status/mood/happiness	Not applicable	Not applicable	Not applicable		Not applicable		Not applicable	Easy to answer but sometimes one cannot hear
ExpoApp	Location, physical activity, Height	Ateknea Solutions, Spain	Not applicable	Depending of Smartphone Battery		Location: 1 Hz Physical activity: 30 Hz Height: 1 Hz		Not applicable	Always works
Global sat, BT335 GPS	Location	GlobalSat Worldcom Corporation, USA	130	18 h		1 Hz		75 g	Easy to wear on spibelt
Actigraph	Physical activity	ActiGraph, USA	200	25 days		100 Hz		19 g	Easy to wear on spibelt
Lascar EL-USB-2-LCD	Temperature, relative humidity	Lascar Electronics, United Kingdom	75	1 year		0.1 Hz		46 g	Easy to wear on backpack
CESVA DC112	Noise	Vertex, Spain	2500	20 h		8 kHz		361 g	Not applicable
Sunbuddy	UV	Bitsplitters, Switzerland	300	4 months		<1 Hz		20–50 g	Pin system does not work well. Looses Bluetooth connection often
Microaetholometer	Black carbon	AethLabs, USA	5900	24 h *		1 Hz		280 g	Not applicable
Paper and pen	Travel destinations	Not applicable	Not applicable	Not applicable		Not applicable		Not applicable	Easy to forget
Backpack	Not applicable	CREAL, Spain	50	Not applicable		Not applicable		1200 g	Easy to wear or put in room, difficult if one has other backpack or does intensive activities
Batteries Energizer Energy box 8000 mAh	Not applicable	ENIX ENERGIES, France	40	25 h		Not applicable		230	Not applicable

Note: * With extended battery.

**Figure 1:**
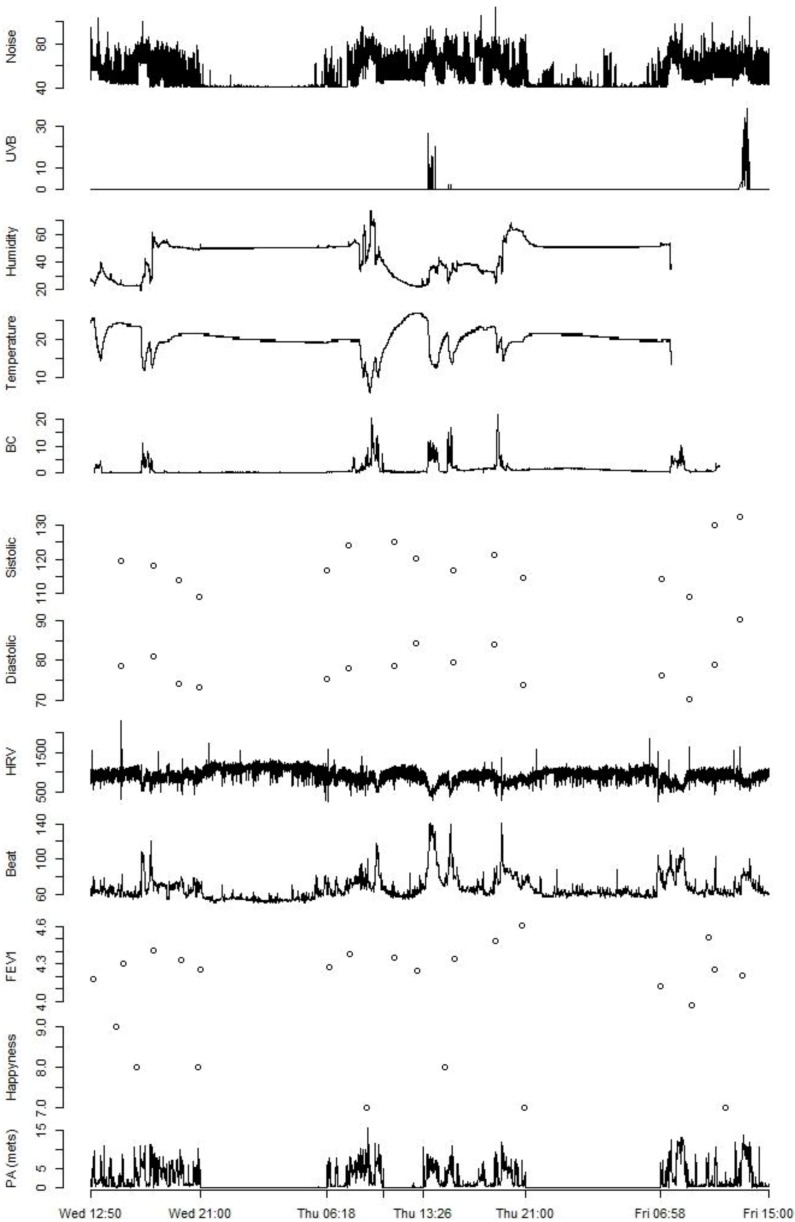
Personal levels of noise (dBA), UVB (mJ/cm^2^), humidity (%), temperature (°C) and black carbon (μg/m^3^) and blood pressure (mmHg), heart rate variability (ms), heart beat (beats/min), lung function (L), emotional status, and physical activity (METs) during two 24 h periods.

Figure 1 shows the measurements of noise, UV, humidity, temperature and black carbon and blood pressure, heart rate variability (ms), heartbeat, lung function (FEV1), emotional status, and physical activity during two 24 periods in November 2013 in Barcelona.

**Figure 2:**
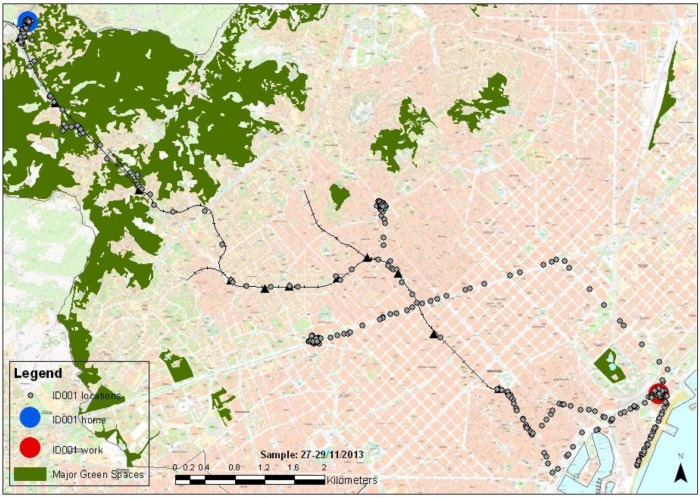
Trips made during two 24 h periods.

**Figure A1:**
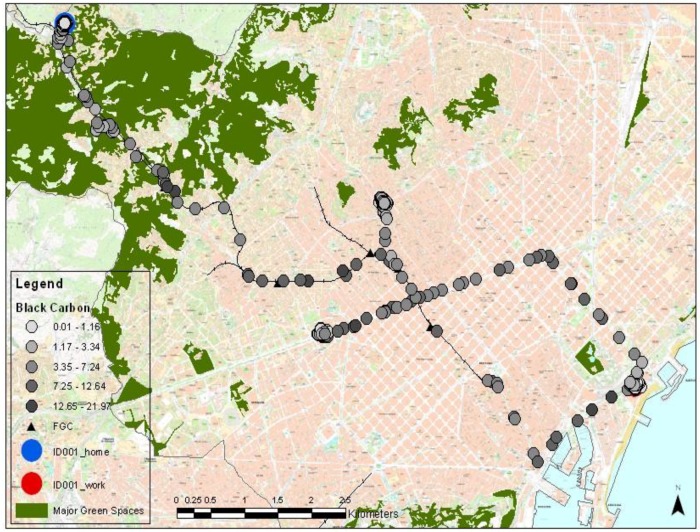
Black carbon levels for one 24 h period at different locations visited.

**Figure A2:**
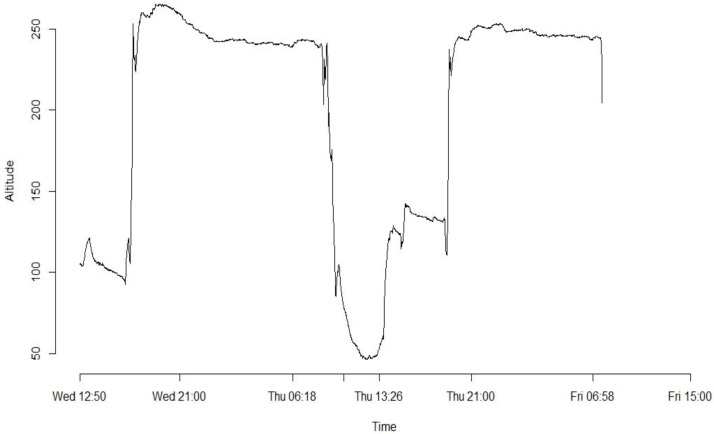
Relative height during two 24 h periods.

**Figure A3:**
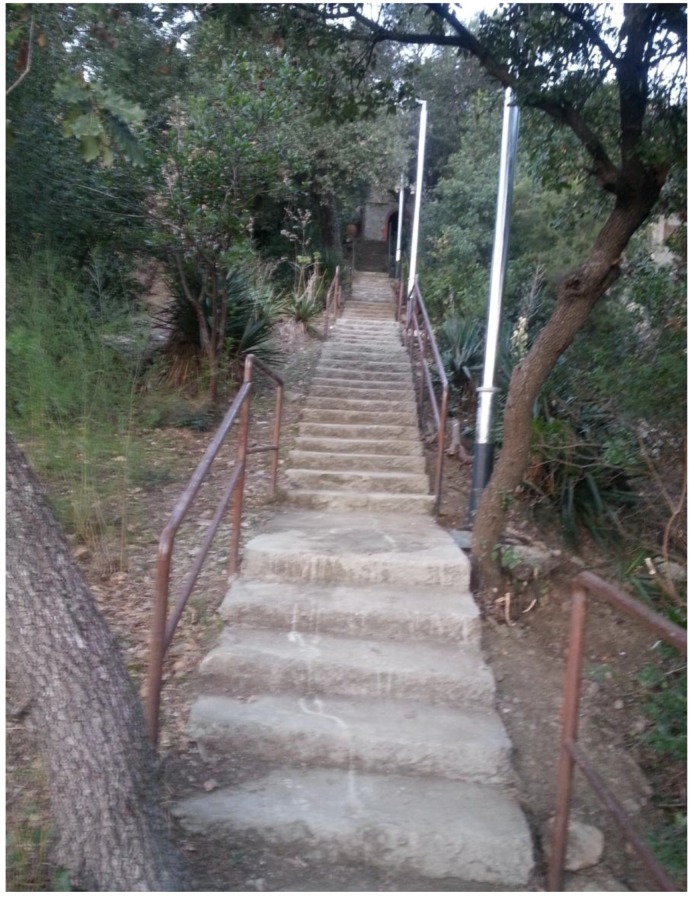
Green space visited.

**Figure A4:**
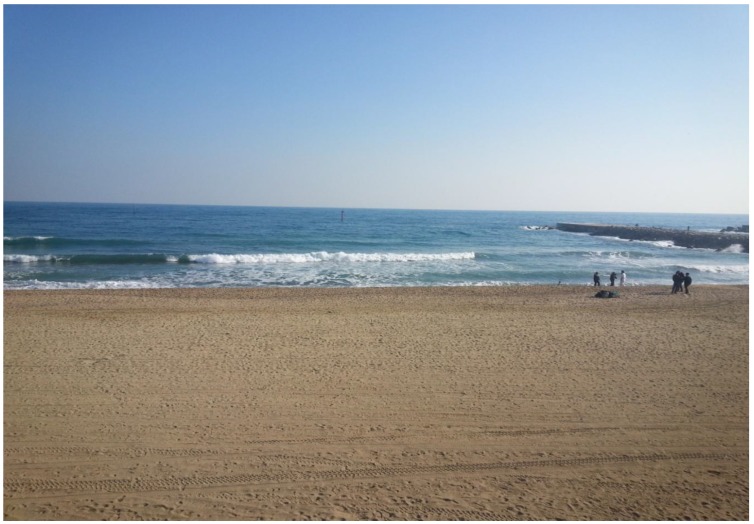
Blue space visited.

**Figure A5:**
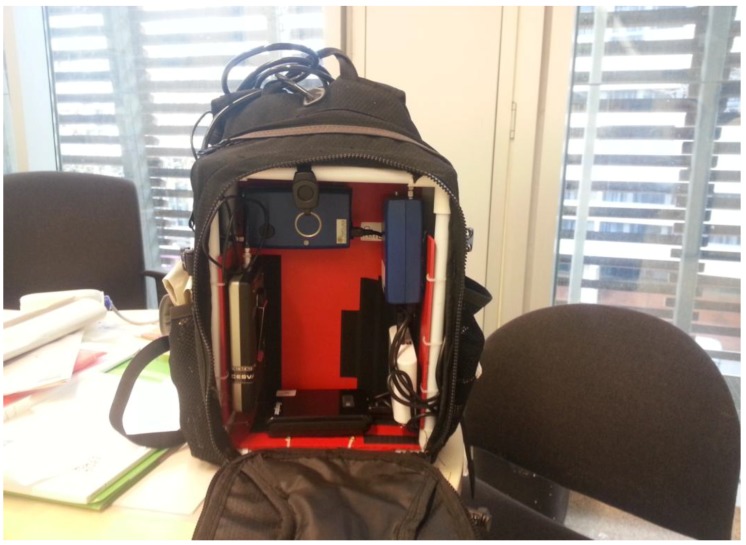
The back pack with equipment.

They show considerable variability for all the parameters measured and some clear differences between day (e.g., higher exposure levels and variability) and night (lower exposure and variability) and indoor and outdoor (e.g., peak exposures for UV, black carbon, changes in humidity and temperature) for the environmental exposure parameters, and patterns of exposure that appear to be correlated. Also peaks in heart beat are shown when cycling outdoors. Figure 2 shows the location of the person during the two 24 h periods. Further information that was successfully collected included the levels of black carbon at specific location, visits of green space and blue space and the altitude of where the person is (Appendix Figures A1–A5).

## 3. Discussion and Conclusions

Personal sensors to measure environmental exposures have been used and are now being used in a number of projects including ICEPURE, TAPAS, PHENOTYPE, HELIX, EXPOsOMICs and HEALS. We demonstrated further the availability and use of personal sensors to obtain information on multiple environmental exposures and acute health effects, but many challenges remain to extend the use to a larger number of subjects.

The ease of wear and operability by a person is one of the key criteria for future use. The current set is probably only suitable for highly motivated people. The back pack with the sensors was bulky, partly as a result of the size of the noise and air pollution sensors and the need for extra batteries to make the sensors run for at least 24 h, and preferably longer. A concern here is that people may change behavior as a result of the size which is to be avoided. Further miniaturisation and increased battery power is needed for some sensors (e.g., for air pollution and noise) to make it feasible to use in large populations, and for extended hours. The eMotion HRV monitor, Actigraph accelerometer, Sunbuddy UV dosimeter and Lascar temperature and humidity monitor can run for more than 7 days without charging and are small which make them easier for this type of use. Also the mini Piko-1 spirometer and OMRON M10 sphygmomanometer record a large number of attempts which makes them suitable for long term use. 

The UV, temperature and humidity sensors are light and were fairly easy to wear and use, but there are concerns such as that the UV sensors may get covered by clothing, although less likely during sunny periods, and that temperature and humidity sensors may get too close to the breathing zone and skin and therefore not measuring the external environment. The quality of the measurements is still an issue for some of these sensors, although we tried to find the best in their field and the ones we used probably provide good data. However some measurements such as those of lung function and blood pressure may not be as reliable as when they are supervised by a trained person and this should be taken into account when analyzing the data. More measurements may be needed to get the same precision as when using a trained person. Furthermore the Microaetholometer AE51 measures only black carbon, which is only one component of air pollution, albeit for which health effects have been reported, and may therefore not be representative of all air pollution. It was used here because it is one of the few small continuous air pollution sensors that measures reliably. Also the CESVA noise dosimeter measures a whole spectrum of noises/sounds (1 octave bands) and further work is needed to differentiate types of noise and sounds, e.g., from traffic, voices, or birds to make optimal use of the data, e.g., by filtering certain frequencies. Even higher refinement in noise frequency measurements (*i.e.*, devices with 1/3 octave bands) would be desired, however, to our knowledge, it is unlikely currently to find high quality noise devices that can measure environmental noise levels (starting at least as low as 40 dB) and that also combine noise spectra. Also, this lower limit of detection still poses a challenge as to measure indoor noise levels, particularly at night, when levels may be below 40 dB indoors, but be potentially relevant for sleep impairment and health. Finally a number of the instruments (e.g., CESVA noise monitor, Microaetholometer AE51) are still too expensive for widespread use and cheaper sensors are needed to allow large scale follow ups. 

We used a smartphone for a number of functions including taking photos, e.g., of green space, but it is easy to forget to take these. Photos can provide a lot of information, but considerable effort is needed to translate photos into useable information for research [40]. We also assessed emotional status/mood at random during the day via smartphone, following the Ecological Momentary Assessment (EMA) principles [41,42]. The method provides great opportunities to collect various responses such as mood and stress levels during the day. The drawback is that at times the subjects do not hear the ringtone and miss the request or are too busy with an activity to respond. It may also be more useful if it can be programmed to alert the subject in specific locations (e.g., green spaces) or during certain activities (e.g., eating). The smartphone was given and easy to wear on the SPIbelt, but in the future most of the applications could be incorporated into the smartphone that people use daily (as opposed to one given on a SPIbelt), except for the physical activity assessment, which generally needs special placement on the waist to obtain valid measurements. A small external accelerometer (e.g., ActiGraph) placed on the hip may therefore be better for physical activity assessment, partly also because they can be run for longer durations. Furthermore wrist based sensors containing accelerometers have come on the market which may also provide a good measure of physical and other activities and may be easier to wear [43].

The combination of information from various parameters can improve the exposure and dose estimates. The assessment of both physical activity and personal air pollution levels allows the estimation of inhalation dose levels [44], which could improve the exposome estimates for the subjects. The combination of information on location and physical activity allows the assessment of where exactly physical activity takes place and whether it may be due to some features such as green space. Furthermore the combination of accelerometry and heart rate data allows a better estimation of the amount of physical activity performed.

As for this occasion, data were downloaded manually after 24 h, but further improvements of the sensor set could have the smartphone communicate with the sensors and send the data on regular intervals to a central server. This is developed, e.g., in the CITI-SENSE project. Furthermore, the sensors can provide large amount of data that need further cleaning and processing and also may need new ways of statistical analyses of the data.

Rather than personal sensoring, an alternative approach could be to create a dense network of embedded ambient sensors [23,36,45,46] where environmental exposures such as air pollution, noise, temperature and UV can be measured and/or estimated at small spatial and temporal scales and then combined with information on mobility and physical activity of the person from, e.g., smartphones to obtain personal estimates [29,37]. The estimates could then be validated with personal sensoring data. The advantage of this approach is that the likely costs could be much lower, it is less burdensome to the subject and that (outdoor) estimates for a larger population could be obtained. The disadvantage is that it may be hard to model the total personal exposure since, for example indoor air pollution and temperature would not be measured, and personal UV depends not only if a person is outside but also whether he or she is in the shade or not. Assumptions would have to be made for these. Furthermore it may need a large network of embedded sensors.

We have discussed and demonstrated the recent developments in personal sensoring of multiple exposures and acute health responses, but the challenge will be to scale up the work and conduct large studies with many subjects to assess the relationship between multiple environmental exposures and physiological, social and psychological measures. This will create a large dataset with multiple exposures and health responses, which cannot be analysed using simple regression models examining one health outcome and one exposure adjusted for a number of covariates and therefore further developments are also number in statistically techniques similarly to what we have seen for the analyses of OMICs data. 

Although we included a considerable number of sensors, there are sensors for other exposures such as EMF [47] or other personal sensors for, e.g., air pollution or noise [23], as there are sensors to measure outcomes such as EEG [48]. Furthermore future use of implanted biosensors for environmental exposures may make it possible to sensor internal doses in real time like is currently done for, e.g., glucose levels [49], physiological parameters [50] and brain activity [51]. Finally, the technological world changes really fast and new technologies such as Googleglass [52] and smart watches [53] may provide further information. Smartphones are each time faster and provide new functionality and build-in sensors and technologies like Bluetooth 4.0 that provide the opportunity to create small sensors with large battery life making it easier to sample new exposures that until now were not practical to measure. 

The aim of this paper was not to provide a fully validated sensor set and/or a dataset using sensors, but to provide a vision of the future and the challenges that remain when using sensors to measure multiple environmental exposures and acute health responses. The challenges are great but technology moves fast and could be used to great advantage to conduct environment and health research. We showed a first finger print of the part of the exposome that could be obtained by sensors, but much more data is needed to provide a whole picture. The challenge is out there.
